# Speaking Softly and Listening Hard: The Process of Involving Young Voices from a Culturally and Linguistically Diverse School in Child Health Research

**DOI:** 10.3390/ijerph18115808

**Published:** 2021-05-28

**Authors:** Nora Samir, Antonio Mendoza Diaz, Michael Hodgins, Simone Matic, Samira Bawden, Jessica Khoury, Valsamma Eapen, Raghu Lingam

**Affiliations:** 1 Population Child Health, School of Women’s and Children’s Health, Faculty of Medicine, UNSW, Sydney 2031, Australia; michael.hodgins1@unsw.edu.au (M.H.); r.lingam@unsw.edu.au (R.L.); 2 BestSTART-SWS, South Western Sydney Local Health District, NSW Health, Sydney 2031, Australia; a.mendozadiaz@unsw.edu.au (A.M.D.); v.eapen@unsw.edu.au (V.E.); 3 Academic Unit of Child Psychiatry, School of Psychiatry, Faculty of Medicine, UNSW, Sydney 2170, Australia; 4 NSW Department of Education, Parramatta 2150, Australia; simone.vavayis@det.nsw.edu.au (S.M.); samira.bawden@det.nsw.edu.au (S.B.); jessica.khoury14@det.nsw.edu.au (J.K.)

**Keywords:** community and consumer involvement, youth engagement, mental health, participatory action research, child health

## Abstract

The involvement of young people in the planning of research continues to be rare, particularly for young people from culturally and linguistically diverse communities. This paper describes our experience in establishing a Youth Research Advisory Group (YRAG) in South West Sydney (SWS), including barriers and successful strategies. One hundred and fifteen students between school Years 7 and 12 (ages 11–18) took part in at least one of five sessions between 2019 and 2021. In total, we carried out 26 YRAG sessions, with between five and 30 students in each. Sessions focused on mapping the health priorities of the participants and co-developing research project proposals related to their health priorities. Our work with students revealed that their main areas of concern were mental health and stress. This led to material changes in our research strategy, to include “Mental Health” as a new research stream and co-develop new mental health-related projects with the students. Important strategies that enabled our research included maintaining flexibility to work seamlessly with organisational and individual preferences, and ensuring our processes were directed by the schools and—most importantly—the students themselves. Strategies such as maintaining an informal context, responding rapidly to student preference, and regularly renegotiating access enabled us to engage with the students to deepen our understanding of their experiences.

## 1. Introduction

Community and consumer involvement (CCI) is increasingly incorporated into health research; however, the inclusion of young people in all aspects of research—from research design to implementation—continues to be rare [1]. The view that engaging end users is important emerged from a paradigm within organisational, leadership, and healthcare scholarship, recognising the value of lived experience [2,3]. CCI in research has been defined as actively involving consumers (i.e., the target group for an intervention, policy, or other output that is being designed), and the wider community in research projects to help identify what should be researched and how research should be carried out to develop solutions that meet consumers’ needs [4,5,6,7]. Benefits of client or consumer engagement are well documented in the health and social care field more broadly, with service innovations to address consumer priorities being a key advantage [8,9,10,11]. The engagement of young people in research and translation is also an Australian national priority [12].

Until recently, published literature on CCI to date has typically involved adult participants [1,13,14]. The lack of youth engagement in CCI has been due to the challenges of navigating gatekeepers such as parents, schools, and ethics committees, and the often-misjudged perception that young people are unable to critically understand and contribute to research planning, implementation, and evaluation [15]. Although research about children and young people abounds, only a minority of the research done with children and young people has involved them in the design, analysis, and dissemination of research [1,16,17,18,19].

In recent years, there has been greater recognition of the value of incorporating youth voices and perspectives into the research planning and implementation process [14,20]. Researchers and policy makers now acknowledge that children and young people have insights into their health and wellbeing that adults do not [21]. Therefore, young people are in the best position to identify areas for improvement or change within the health system. As one girl in an Australian participatory study said:

“Kids should be asked about stuff that’s got to do with them … They can tell you stuff you’d never think of—cos you’re not a kid …” (Girl, aged 7; Involving children and young people in research, 2008).

Although there is growing interest in this area, there are limited examples describing the pragmatic and operational aspects of meaningful engagement with young people in research. This is particularly true for children from culturally and linguistically diverse communities. This paper addresses this gap by describing our experience in establishing a Youth Research Advisory Group (YRAG) in South West Sydney (SWS), including barriers and successful strategies in the hope that they will be useful for others trying to integrate the voices of children and young people in their work.

## 2. Materials and Methods

This initiative was established through the academic group BestSTART-SWS (Systems Transformation and Research Translation), a translational research group established in 2019 to optimise the health, development, and wellbeing of children in SWS. One of BestSTART-SWS’s key aims was to integrate the voices of children and young people (CYP) from the local area into planned and current research efforts to better meet the needs of the community. This article will focus exclusively on the involvement of CYP from local schools.

The YRAG was based in the South Western Sydney Local Health District (SWSLHD), which is an area with a population of 966,450 characterised by its cultural diversity and economic disparity. Approximately 1.6% of the district’s population identified as Aboriginal or Torres Strait Islander, 44% of the population is born overseas, and 51% of the population reported speaking a language other than English at home (SWSLHD, [22]). The most common local languages were Arabic, Vietnamese, Spanish, and Cantonese. The district is also a major point of settlement for refugees with approximately 10,932 refugees—41.7% of all refugees in NSW (2005–2011). The population also experiences large social and economic disparity, with some of the poorest communities in NSW as measured by the socio-economic index for areas (SEIA) in 2016, and a higher rate of disabilities than the state average (SWSLHD, [22]).

### 2.1. Ethics

Ethical approval for this study was granted by the UNSW HREC (HC190441), the SWSLHD HREC (2020/ETH00149), and the SERAP HREC (2020013). Full explanation of the ethical nature of the study is explored in the section “Approaching the schools and setting up the groups” below.

### 2.2. Participants

One hundred and fifteen students (15 males and 100 females) from two single-sex schools took part in at least 1 of 5 sessions as part of the YRAG between 2019 and 2021. The age of participants between school years 7 and 12 ranged from 11 to 18. Sessions were organised flexibly, such that in one school they were organised by year group, while in another, only one age group (Year 12) was involved. In total, the team carried out 26 sessions, with between 5 and 30 students in each. All students that initially agreed to participate attended all of the YRAG sessions they were invited to, unless they were absent from school on that day.

### 2.3. Data Collection

Sessions lasted between 1 and 1.5 h and took place at either participating schools or the research facility hosting the BestSTART-SWS research group. Session frequency varied from 3 months (initially) to 9 months (during the COVID-19 pandemic). The series of sessions focused on five areas: (1) defining health and mapping the health priorities of the participants, (2) priority setting to refine the health priorities of the participants, (3) co-development of research project proposals, (4) exploring existing research in the area, and (5) provision of feedback on the impact of the CCI work. Sessions are continuing to be organised and have been iteratively adapted based on participant feedback, for example, by focusing on specific themes raised by the priority-setting activities. Continued engagement allows for the establishment of meaningful relationships with young people and the community, which are seen here as a goal in itself. This is in opposition to engagements based on single research projects, which are often more transactional and transient in nature. In addition to the data from sessions, the facilitators also kept a journal noting the process of establishing the sessions and noteworthy experiences along the journey. They also recorded observational field notes, reflecting on how the sessions went and how participants responded, noting their levels of engagement with different topics and facilitation techniques.

The facilitators were both young adults, aged 29 and 24, one male and one female. AMD was born in Venezuela, and NS was born in Australia and has an Iraqi background. AMD is a provisional psychologist in child mental health and NS is a population child health researcher trained in public health. Both have received mental health first aid training. Both AMD and NS hold socially liberal views. Their views and opinions were, for the most part, withheld from participants; however, we acknowledge these biases in our current perspective.

### 2.4. Data Analysis

Our analytic approach involved reviewing session data, field notes, and journals by the authors; the development of lists of key themes, guided by extant literature on youth participation in research; and discussions with other authors for the development of a thematic structure. The thematic structure was guided by a narrative approach, providing a chronological perspective on our entry into the site and undertaking of consumer and community involvement with young people.

## 3. Themes

### 3.1. Approaching the Schools and Setting up the Groups

The researchers responsible for organising the CYP engagement strategy (i.e., the authors of this paper) believed that engagement should happen “on their terms”, meaning that the format (e.g., number/time of meetings, number of participants) and characteristics of the group should be jointly determined with CYP and their schools. This view allowed the team to flexibly approach stakeholders and start discussions based on shared principles (e.g., “Listening to young people is important”) rather than negotiating predetermined demands (e.g., “We need 10 students for this group”). Although this helped progress some forms of access, such as in engaging school administrators, it was less helpful in navigating ethics approvals, which is a topic covered separately below. All school administrators approached agreed with the research team on the importance of giving CYP a voice in research about them. From the three schools approached in 2019, two have gone on to establish the YRAG, while the third is yet to grant researchers access to its students, despite repeatedly expressing a keen interest to participate.

#### 3.1.1. Strategies in Approaching Schools

The team enacted several strategies to approach school principals, with some of these strategies helpful in negotiating access and others less helpful in enabling the team to continue its work. Helpful strategies included addressing common values between researchers and school administrators, “delegations” made-up of both senior and young researchers (representing both the authority and the enthusiasm of the team), flexibility in the terms and conditions of the YRAG, attending meetings at the principal’s office/school, clarity around expected benefits of the engagement for all parties (i.e., students, school, researchers) and, later on, ensuring lines of communication remained open, providing feedback on the group’s progress, and directly addressing concerns with administrators and parent groups. In contrast, unhelpful strategies included diffusing the invitation to participate in the YRAG by mentioning other research projects that the school could be involved in and uncertainty—for both schools and researchers—regarding who were the key decision-makers (e.g., school principals, vice-principals, or community liaison officers). It was important as part of our engagement strategy to incorporate a flexible approach with rigorous planning. We wanted to avoid the incorrect perception that our lack of a rigid predetermined structure for the project was due to disorganisation or flippancy. To avert this perception, the team attempted to communicate to stakeholders the importance of the work first (e.g., “our main priority is to include the students in consumer involvement research priority setting, but how we do that is the focus of this discussion”) to help establish a shared vision on how best to facilitate the project. Other strategies used to build trust with schools included attending school assemblies and events (e.g., the science fair), organising a night for parents to speak with researchers (although this was scarcely attended), and inviting school staff to the BestSTART-SWS strategy day in a show of camaraderie.

#### 3.1.2. Strategies to Approach Students

The differences between schools were reflected in their suggestions regarding how best to approach their students. Two of the schools invited researchers to present their plan at their school assemblies, where all students gathered to listen to the day’s announcements. The team was initially wary of engaging the whole student body as this could come across as impersonal, and it was expected that garnering their interest in research might have needed a more individual approach. Researchers gave a similar pitch at two different schools (e.g., “We want to make sure that what is important to you [students] is taken into account in our research”; “We don’t want to do research about you without you”), which generated very different outcomes. At one school, the assembly occurred during lunch, when researchers were introduced warmly by the school principal and spoke to the student body from a pulpit with a microphone. At another, engagement with students felt hurried and decontextualised. Perhaps predictably, when researchers felt most supported, the recruitment drive was a lot more successful, with recruitment varying by an order of magnitude (10s vs. 100s) between these schools. Other strategies that proved helpful in engaging young people included shared demographic characteristics between researchers and participants (e.g., age, culture, gender), appealing to young people’s desire for change (e.g., “This is something that research hasn’t done well; we need to change that”), being open about the team’s areas of expertise as well as their blind spots (e.g., “I don’t know what’s going on for you”), and—perhaps most importantly—allowing them to participate in the group during class time (instead of being in class). Feedback from students on these strategies was positive, with students commenting the sessions were a welcome relief from daily routine, while noting they would be unlikely to participate if groups were organised outside of school hours. Alongside these strategies, the team also mentioned the benefits of participation, such as personalised certificates of involvement, references, and snacks. Some of these ancillary benefits to students changed depending on what was permissible at each school.

#### 3.1.3. Strategies in Managing Regulatory Approval

Navigating access to CYP was difficult as health-based researchers, as approvals were sought from NSW Health, the Department of Education, and the university. In total, it took over two years for approval to be granted. Fortunately, the research project had already begun as “consultations” with schools and students, and this helped the team expedite the ethics process. The difference between “consultation” and “research” turned out to be pivotal. Consultation is—for the most part—outside of the purview of ethics boards, and so approval to consult students on BestSTART-SWS’s research strategy was granted much sooner than approval to involve CYP as part of a research project exploring their health priorities. For both consulting students and involving them in a research project, the team was required to generate Working with Children Checks, Police Checks, Statements of Confidentiality, etc. An important distinction was that the content of consultations needed to remain internal (within BestSTART-SWS), whereas the content of a research project is intended for wider distribution. This was a challenging decision, as the team could either focus on the ethics approval, without knowledge of how long this would take, or it could start by working with CYP while knowing that the outcomes would not be publishable. This team decided to enact the latter strategy and engage young people in initial consultations that then supported the ethics application.

In this respect, client-led work is ill-suited to the existing prescriptive ethics architecture, which perceives deviations from an initial plan as potential risks, rather than as a natural part of the dialectical process we intend it to be when engaged in CCI work. Adequately addressing this issue is a matter for funding and regulatory agencies and is beyond the scope of the strategies presented here. However, some strategies that could be of use for future projects may include meeting with the ethics committee representatives to discuss how best to seek approval for the study, ensuring the protocol and ethics application are described with room for variation (e.g., strategies and sessions are described broadly), and consulting with stakeholders before ethics approval is sought, such that reviewers understand all parties agree to take part. Finally, it is highly likely that some reticence in engaging CYP as co-designers is due to the complex and time-consuming process of seeking ethics approval, and this should be seen as a risk for the promotion of CCI work with CYP.

### 3.2. Establishing and Running the Groups

At the conclusion of the engagement process, two schools elected to take part in the YRAG. One had roughly 100 potential participants and the other had 15; both models were accommodated. At the first school, the young people were divided by age-group/grade, whereas in the second, the 15 participants were members of a student leadership body made up of select individuals who were thought to be good representatives of the student body. The differences in composition reflected the fact that a lot of students had signed up to take part in one school, whereas in the other, only members of the student leadership body had chosen to take part. These schools service similar populations in terms of their sociodemographic characteristics; however, the school with poor recruitment was an all-boys school, while the other school was exclusively for girls. This section describes the processes that helped foster trust with students—allowing them to fully engage with the content—the methods for (re)negotiating school access, and the results of providing iterative feedback as part of a collaborative approach.

#### 3.2.1. Fostering Trust with Students

When facilitating and managing the YRAG’s, the team had to ensure that students felt safe, trusted, respected, and most importantly, that their voices were heard. The process of introducing the group as part of the first session was standard: introductions, ground rules, an ice-breaker activity, and brainstorming. However, care was taken to ensure the students felt valued (e.g., they were thanked for their time, we remembered their names), and to foster an ambience of enthusiasm (e.g., big smiles, rhetorical questions such as, “are you ready?”, disclosures of emotion such as, “We’re excited to be here with you.”). There were also a few conscious decisions that helped separate this work from their normal “school” work and “school” rules. This was important, as we did not want the students to share what they perceived to be “correct”, but what they were actually feeling and experiencing. To do this, we allowed the use of mobile phones throughout the sessions, as long as participants respected others who were talking. We also ensured the sessions felt informal by asking students to call us by our first names, incorporating humour in our discussions (e.g., GIFs and current memes in slides), and using active listening strategies to demonstrate engagement. These strategies were effective in breaking down barriers between researchers and students. For example, students were unlikely to actively disengage from sessions, instead bringing in content from their lives outside of school into the discussion (e.g., by searching for information; sharing videos or memes that expressed how they felt about specific topics).

The facilitators’ (NS and AMD) age and background was another contributing factor to the establishment of trust with the students. In a reflection following one of the sessions, AMD noted the importance of NS’s presence for the young women in particular: *“The girls saw themselves in NS, and this was a big part of why we had a massive response to our visit today”.*

Where appropriate, we talked about our personal lives and our own experiences as well. For example, NS spoke about her kindred experience with many of the young women present, how her family had immigrated to Australia from Iraq 24 years ago, and how this inspired her to pursue research and dedicate her life to improving the lives of culturally and linguistically diverse populations. While we appreciate that finding a member of the research team that young people can identify with is unrealistic for most research teams, the importance of acknowledging the real and challenging experiences of participants is vital in fostering trust, perhaps even more so for young people.

#### 3.2.2. (Re)negotiating School Access

The process of engaging CYP in CCI work involves renegotiating access at different points in time. This might happen because of staffing or role changes, administrative decisions, school requirements, or a global pandemic. Here, we discuss instances in which access to CYP had to be re-negotiated with schools, some of the strategies employed, and what this meant for our relationship with schools.

At the start of the YRAGs, we negotiated teacher presence in the rooms. Schools required that a teacher/chaperone be present. This was something that neither the researchers nor the students wanted, as it might inadvertently censor the discussions, particularly when these turned towards ways in which the schools could better service the health needs of their students. This was discussed with the school and they were very sensible, deciding to allow teachers to sit just outside of the room. In this way, students were comfortable to speak their thoughts with the knowledge that their involvement would not impact on their relationship with the teachers and school. The team was also fortunate enough to have access to glass rooms that allowed teachers to see, but not hear, the discussions inside the room. During the COVID-19 pandemic, we were unable to conduct the YRAGs in person, as visitors were not allowed in the school as part of the pandemic protocol. Whilst we were able to conduct one session online to stay in contact with the students, the online format did not work very well and was difficult to manage, as students spoke at the same time, and it was hard to personally relate to the students. Interactions felt impersonal, despite existing relationships with students, and access needed to be re-negotiated with schools post-lockdown. In some ways, this was a step back for the relationship building, but in other respects, it meant a shared experience of living through a real crisis, which may cement trust in the relationship going forward, as discussed below in the “future directions” section.

### 3.3. Exploring Health Priorities: The Emergence of Mental Health as a Priority Area

As mentioned earlier, the initial engagement with students was organised to share BestSTART-SWS’s research strategy document with students to ensure that it met the priorities of the community of children and young people in the area. This strategy included four research streams based on the expertise of the research team: healthy beginnings, population child health, health systems research, and equity. Consultations with children and young people revealed that their main areas of concern—particularly when thinking about health—were mental health and stress. In response to this realisation, BestSTART-SWS adapted its research strategy to include “Mental Health” as a new research stream, recruiting experts who could lead this stream and ensuring students remained engaged in determining the kinds of activities the stream would be responsible for. The details of discussions with students and the arrival at the “Mental Health” topic is the subject of a future article; however, a number of exercises trialled with students bear mentioning in this piece, as they differed in their success. In the section below, four exercises are detailed alongside reflections on their effectiveness.

Some of the exercises developed to help in the priority setting work with students included brainstorming, two-alternative forced choice (2AFC), list-ranking, and case study analyses. Priority-setting activities began with thorough brainstorming, in which facilitators used butcher paper to generate mind maps while promoting an open-ended discussion focusing on breadth (e.g., “It seems we have listed several types of physical health problems, what else comes to mind when we think about health?”). This approach worked well because ideas generated canvassed multiple areas, touching on different aspects of health. A weakness of the brainstorming activity was that some participants felt discussions were at times led by the most vocal group members. These vocal group members sometimes steered the group in directions not all participants agreed with, despite the facilitators’ best efforts. To address this, facilitators introduced post-it note consultations when sensitive topics were raised that demanded differences in opinion. As an example, after a discussion on different mental health strategies, facilitators asked the students to write down on post-it notes which of the strategies they felt would “work for them personally”. This allowed us to examine differences in opinion between group members rather than taking the vocal majority at face value.

**Figure 1:**
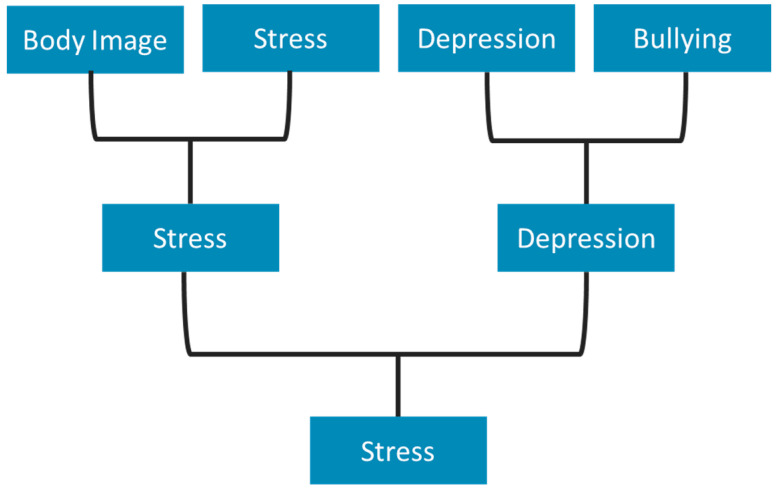
Example of a voting bracket, as proposed for session three.

2AFCs were organised with students after brainstorming had generated a plethora of different topics that the students felt were important. Here, students were asked to vote on which topics felt most personal and relevant to them. 2AFCs were organised as a sport bracket, such that the winner of Pair 1 would be compared to the winner of Pair 2, and so on until a single topic was selected. An example of these brackets can be seen below in Figure 1. Importantly, the categories used had been generated by the group itself as part of our discussion, and the “seeds” (or original pairings) were alternated between groups to ensure that starting conditions were not responsible for the results. This exercise seemed to work well, as the students enjoyed voting and were at times surprised by the final results. Facilitators kept an upbeat pace when discussing these comparisons and projected a spreadsheet that filled out the contenders based on which topics had “won”. At the end of the exercise, facilitators engaged the group in open-ended discussions in which the validity of the exercise itself and the results were questioned with students to ascertain whether these results reflected their experiences. Students seemed to agree that the exercise had been worthwhile.

Another one of the exercises attempted involved list-ranking wellbeing strategies aimed at addressing a specific topic, such as “stress”. Here, there was an initial presentation by facilitators that provided background information on what was involved for each strategy (e.g., mindfulness, cognitive challenging, active listening), after which students were given cards listing each strategy and were asked to rank them by placing preferred strategies at the top and least-preferred strategies at the bottom. Students completed their individual lists, but their clarifying questions and overall level of engagement suggested that they were not connecting with the material in the way we had hoped. With the benefit of hindsight, it seems clear now that it is unlikely that students would have formed a strong preference for a strategy that they had never heard about. As before, at the conclusion of the activity, the exercise was discussed with students and, confirming our suspicions, students mentioned feeling confused at the differences between some of the strategies and uncomfortable at the lack of personal experience with the content being discussed. While list-rankings such as these have a place in consultative work, it is likely they are more fruitful in contexts in which young people are allowed to be the experts (i.e., can speak about their own experiences), rather than be asked to make judgments on content that is largely outside of their personal experience.

Lastly, the discussion of case studies with participants proved to be a fruitful strategy. The case studies described evaluations of school-based mental health interventions that were edited by facilitators into brief and digestible formats. Students enjoyed learning about what had been trialled and had a lot to contribute. It is interesting to juxtapose these discussions with the list-ranking strategy mentioned above, as in both cases, students were asked to consider information that was outside of their own experience. However, unlike with the list ranking, with the discussion around concrete examples, students were able to identify those elements that resonated with their own experiences and direct the discussion to those areas that felt most personally relevant. For example, when discussing an intervention that involved yoga and mindfulness classes before the start of school, students expressed scepticism at whether this would work at their school (e.g., “This wouldn’t work here miss, no one would come early to school for yoga.”). This was different to discussions around “mental health strategies” which had been abstract, depriving students from the opportunity to relate to these personally.

## 4. Discussion

This paper describes our experience in establishing a YRAG in South West Sydney (SWS) to aid researchers integrating the voices of children and young people in similar contexts. Important strategies that enabled our research were maintaining flexibility to work seamlessly with organisational and individual preferences, ensuring our processes were directed first and foremost by the schools, school staff, and, most importantly, the students themselves. In combination, strategies such as maintaining an informal context, responding rapidly to student preference, and regularly renegotiating access enabled us to engage with the students to deepen our understanding of their experiences. Our study reflects the growing youth-oriented CCI movement, which indicates that engagement with young people is essential to developing grounded and coherent research strategies [1,23]. Current research approaches and methods need to be more encompassing of the diversity of children and childhoods, which is an observation that has been consistently stated by scholars in the field [24,25,26]. For example, Involve UK has published minimum standards for good practice in engaging children and young people; however, contextualisation for different settings, such as the work detailed in this paper, is needed. Many children’s voices remain unheard, particularly children living in marginalised and vulnerable communities, such as those in culturally and linguistically diverse populations [18].

Engagement in research and health decision making can be empowering for disadvantaged populations; however, health traditionally has used ‘top–down’ engagement approaches as opposed to ‘bottom–up’ participatory methods, which limit their impact upon health and health behaviours [27]. Cyril and colleagues [28] conducted a systematic review exploring the role of community engagement in improving the health of disadvantaged populations and found that community engagement can lead to improved health and health behaviours among disadvantaged populations if designed properly and implemented through effective community consultation and participation. The key community engagement components that affected health outcomes included real power-sharing, collaborative partnerships, bidirectional learning, and using bicultural personnel as part of engagement and/or intervention delivery, aligning closely with our findings [28]. Our strategy to ‘speak softly’ to promote the empowerment and engagement of young culturally and linguistically diverse people in their participation in research also aligns with the findings of Ferrera and colleagues [29] from a community-based participatory research project, which showed that the non-hierarchical approach in their programme resulted in social capital and empowerment among immigrant youth in Chicago. Our methods also aimed to limit the institutional power of the school as a context for discussion by providing a space free from teachers’ control and observation, which can change the way young people’s voices are produced [30]. Other similar work exploring the mental health challenges of at-risk young people in the UK has shown that community involvement enables a deep engagement of the issues young people face, as well as the opportunity to create impactful strategies to confront those issues [31]. This work has been projected to have a long-term positive impact and significant improvements in health outcomes for the populations involved [32].

It is vital that researchers consider the importance of the research methods themselves, rather than viewing these methods as just a means to an end to ensure publication and other research outputs [26,33]. The potential positive impacts of CCI methods are wide-reaching, covering all stages of research, including the development of research objectives, questions, documentation, recruitment, analysis, implementation, and dissemination. These impacts are echoed by the National Health and Medical Research Council recognition of CCI’s importance for research, ensuring that research is not just ethical but also relevant and acceptable from the public’s perspective [34]. As shown in our experience, involving children and young people in research has the potential to benefit the quality and relevance of research and the children and young people themselves. Participants involved in CCI have reported that it helped them feel listened to [35,36] and valued [37]. An Australian study in a hospital setting incorporating the voice of young people noted that children felt empowered, listened to, and were able to engage with peers and experts in issues that mattered to them [38]. Additionally, being involved in a research project helped young people add experience to their CV to enhance their chance of finding employment [39]. For young people from disadvantaged communities, these are valuable benefits.

## 5. Conclusions

We consider our client and consumer involvement work with students in South West Sydney to have been successful, as it has steered research strategy towards those topics that students found most important (i.e., mental health and stress). This led to material changes in research strategy and was shared with other groups that followed suit. This included feeding results back to teachers and school principals, who were grateful for the insights generated and expressed their support for the groups. Moreover, the relationships established to create and run the groups are continuing, and students will become co-designers of research projects aimed at addressing their health priorities.

When we started this effort with BestSTART-SWS, we had little guidance, and there were many detractors and gatekeepers that prevented the team from engaging with students. Navigating these challenges in connecting with schools, engaging students, and talking about their mental health priorities has brought about real change in child health research in South West Sydney. At every step of the way, the research team kept a “long-view” of their relationship with schools, as the sustainability of these efforts over time is a key consideration. Indeed, as the socio-cultural landscape changes, it is the team’s hope that engagement with students can continue, as—perhaps more than any other group—they are the heartbeat of the present. It is our hope that these kinds of efforts can be replicated elsewhere to ensure that research is grounded in community needs and experiences.

## Data Availability

The data presented in this study are available on request from the corresponding author. The data are not publicly available to maintain confidentiality of participants.
